# Efficient Dehazing with Recursive Gated Convolution in U-Net: A Novel Approach for Image Dehazing

**DOI:** 10.3390/jimaging9090183

**Published:** 2023-09-11

**Authors:** Zhibo Wang, Jia Jia, Peng Lyu, Jeongik Min

**Affiliations:** 1 Graduate School of Artificial Intelligence, Jeonju University, Jeonju-si 55069, Republic of Korea; wang970128@jj.ac.kr (Z.W.); kimmit628@jj.ac.kr (J.J.); lyupaif@jj.ac.kr (P.L.); 2 Artificial Intelligence Research Center, Jeonju University, Jeonju-si 55069, Republic of Korea

**Keywords:** image processing, image dehazing, deep learning, U-Net

## Abstract

Image dehazing, a fundamental problem in computer vision, involves the recovery of clear visual cues from images marred by haze. Over recent years, deploying deep learning paradigms has spurred significant strides in image dehazing tasks. However, many dehazing networks aim to enhance performance by adopting intricate network architectures, complicating training, inference, and deployment procedures. This study proposes an end-to-end U-Net dehazing network model with recursive gated convolution and attention mechanisms to improve performance while maintaining a lean network structure. In our approach, we leverage an improved recursive gated convolution mechanism to substitute the original U-Net’s convolution blocks with residual blocks and apply the SK fusion module to revamp the skip connection method. We designate this novel U-Net variant as the Dehaze Recursive Gated U-Net (DRGNet). Comprehensive testing across public datasets demonstrates the DRGNet’s superior performance in dehazing quality, detail retrieval, and objective evaluation metrics. Ablation studies further confirm the effectiveness of the key design elements.

## 1. Introduction

Image dehazing represents a key research theme in computer vision. Its primary goal is to retrieve the crispness and detail from images compromised by haze. Its relevance extends across various sectors, including remote sensing image processing, autonomous driving, video surveillance, and image restoration. Turbid substances such as haze, smoke, and water droplets in the atmosphere lead to light refraction, scattering, and absorption during transmission, causing image degradation, obscured details, contrast reduction, and color distortion. This degradation process is typically captured by the atmospheric scattering model [1,2,3]:

(1) I(x)=J(x)t(x)+A(1−t(x))

Here, I is the observed hazy image, J is the latent haze-free image, t is the medium transmission map, A is the global atmospheric light, and x represents the brightness or pixel value of a location in the haze image. In more detail, J(x)t(x) is called direct attenuation and describes the scene’s radiance and decay in the medium. A(1−t(x)) is called airlight, results from previously scattered light, and leads to the shift of the scene color. If the atmosphere is homogeneous, then the transmission t can be represented by $t\left(x\right)=e^{\beta d(x)}$, where β is the scattering coefficient of the atmosphere. It indicates that the scene radiance is attenuated exponentially with the scene depth d. Image dehazing methods aspire to yield the latent haze-free image J, using the input hazy image I directly or indirectly. It is important to note that Formula (1) represents a simplified expression of the Koschmieder model, suitable for small scattering approximation. This approximation applies to a low haze concentration, short light propagation distances, and isotropic scattering. While the simplified scattering model might overlook certain complexities in real-world scenarios, it can suffice for experimental and algorithmic validation purposes. Therefore, it finds widespread application in the field of image dehazing.

**Figure 1:**
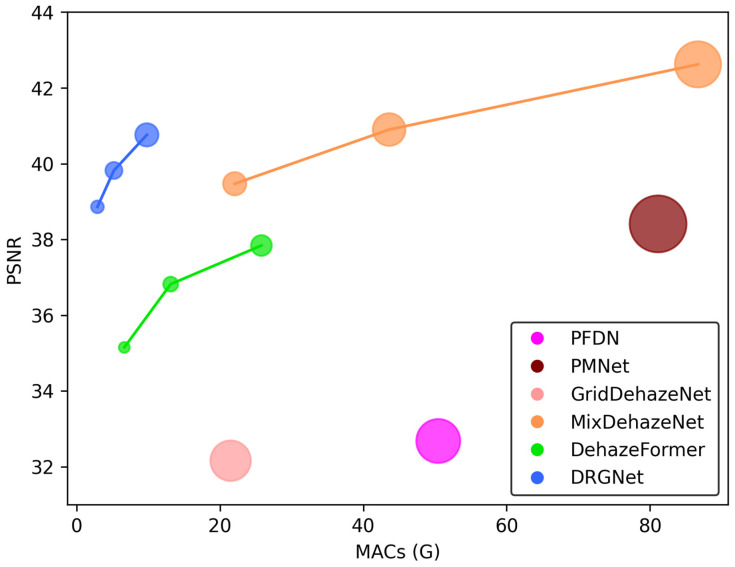
Comparison of DRGNet with Alternative Approaches on the SOTS Indoor Dataset. In this graph, we employ Multiply–Accumulate Operations (MACs) as a metric for quantifying computational load and efficiency across different models. Additionally, we utilize the Peak Signal-to-Noise Ratio (PSNR) as a benchmark to assess the dehazing performance of each model. The size of the data points in our visual representation corresponds to the parameter count of the respective models.

Traditional image dehazing methods, largely dependent on physical models and manually designed priors, often fall short in complex scenes. However, with the advent of deep learning, image dehazing methods rooted in deep neural networks have emerged as a focal research area. Early dehazing networks, primarily based on the atmospheric scattering model, calculated J by estimating A and t values separately. These methods, however, lacked effectiveness in restoring details and textures in intricate scenes. Current advanced dehazing networks deviate from the atmospheric scattering model, employing pairs of hazy and haze-free images for model training and restoring high-quality haze-free images by learning the mapping relationship between hazy and haze-free images. The significant advantage of this approach lies in its capacity to generate haze-free images directly from input hazy images, lessening the dependence on prior knowledge and strengthening model robustness. Despite the remarkable progress in dehazing networks, their growing complexity poses challenges as performance improves. To enhance the dehazing performance while maintaining network simplicity, we integrated minor modifications to the classic U-Net [4] architecture, resulting in a high-performance, lean, compact dehazing model: the Dehaze Recursive Gated U-Net (DRGNet). In particular, we used the classic U-Net as our base architecture, which possesses local residuals [5] and global residuals [6], thereby enabling the extraction of multi-scale image information. We established residual blocks using an improved recursive gated convolution mechanism [7], effectively infusing dynamic weights and high-order spatial interactions into the model, thereby improving feature extraction. We also improved the SK module [8] to embed a channel attention mechanism into the model, enabling the dynamic fusion of low- and high-level features. To validate the efficacy of the DRGNet, we engineered three variants of differing depths and executed trials using numerous prevalent image dehazing datasets. As depicted in Figure 1, the DRGNet delivers superior performance and comparatively lower computational overhead while preserving a lean structure. Additionally, we performed an array of ablation studies to explore the influence of specific design elements within DRGNet on the model’s dehazing outcome.

In summary, our work introduces a lean dehazing network: the DRGNet. Compared to current mainstream dehazing networks, the DRGNet offers an outstanding dehazing performance while requiring fewer parameters and incurring lower computational overhead. A simplified network can be easier to train, infer, and deploy. It also shows that we can still achieve better and better performance through careful design when not using complex architectures.

## 2. Related Works

We can categorize mainstream image dehazing methods into four groups.: methods based on image enhancement, methods based on prior knowledge, methods based on fusion, and methods based on deep learning. In recent years, many research works have used multiple methods simultaneously to enhance performance.

Image enhancement-based methods usually do not use the physical model of haze generation and do not pay much attention to image quality. They use image enhancement technology to increase the visual effect of foggy images, thereby highlighting specific details. Standard image enhancement methods include histogram equalization [9,10], Gamma correction [11,12,13,14], multi-scale retinex [15], white balance method [16], median filtering [17,18], and others. However, dehazing methods based only on image enhancement usually face problems such as pixel oversaturation.

Image fusion-based methods aim to select the best regions from multiple images to synthesize high-quality images. Among these methods, the method proposed in [19] is representative. This method first uses gamma correction coefficients to generate a set of front-exposed images, and then pixel-wise weight maps are constructed by analyzing both global and local exposedness to guide the fusion process. This method is very computationally efficient and generates haze-free images. The color quality is good and the details are precise. In addition, classic image fusion-based dehazing methods also include [20,21], but they have high computational complexity.

Methods based on prior knowledge generally employ physical laws or manually crafted rules to tackle image dehazing. Among them, the Dark Channel Prior (DCP) method [22] stands out as the epitome. The DCP method, premised on physical assumptions, mainly targets outdoor hazy images. Guided by the atmospheric scattering model, it exploits the dark channels within an image to estimate atmospheric light and transmittance, subsequently restoring the haze-free image. Other notable prior knowledge-based methods encompass the Color Attenuation Prior method [23] and the Edge-Preserving Decomposition-Based method [24]. These methods are lauded for their robust interpretability, efficiency, and simplicity, offering promising dehazing results in specific settings. However, they need more adaptability, resulting in a subpar performance in complex environments. These classic prior knowledge-based methodologies have played a crucial role in early image dehazing research, setting the foundation and inspiration for ensuing studies.

On the other hand, learning-based methods utilize deep learning techniques for dehazing tasks, emerging as a research hotspot in recent years. Based on their dependence, these methods can be further divided into two subcategories: those reliant on the atmospheric scattering model and those that are not. Early dehazing networks [25,26,27] typically rested on the atmospheric scattering model, accepting hazy images as inputs, outputting medium transmission maps or global atmospheric light, and restoring latent haze-free images per the atmospheric scattering model. However, in contrast to prior knowledge-based methods, these early dehazing networks needed to exhibit substantial advancements in principles or performance.

As deep learning has matured and our understanding of image dehazing has become more nuanced, the most advanced dehazing networks have veered away from rigid physical assumptions and manually designed rules. Instead, these networks now employ a data-driven strategy, generating haze-free images directly from their hazy counterparts [28,29,30,31,32,33,34,35,36,37]. Depending on the architecture, commonly used dehazing networks, which function independently of the atmospheric scattering model, encompass encoder–decoder networks, GAN-based networks, attention-based networks, and Transformer-based networks. These advanced networks demonstrate superior generalization capabilities and robustness compared to their early predecessors. As a result, they deliver a superior performance in complex scenarios, such as those involving dense and uneven haze, thus asserting themselves as the prevailing approach. Despite their advancements, intricate network architectures pose their challenges. They enhance network performance and significantly escalate the network’s complexity, making training, inference, and deployment more challenging. Balancing performance enhancement and complexity is an ongoing area of investigation in dehazing networks, and this is also the problem we intend to solve in this paper. In addition to the methods based on supervised learning introduced above, some researchers have proposed defogging methods based on unsupervised and semi-supervised methods. Ref. [38] proposed a method to continue unsupervised learning, solely using real-world outdoor images and tuning the network’s parameters by directly minimizing the DCP. Ref. [39] proposes a semi-supervised learning network: SAD-Net. SAD-Net utilizes both synthetic datasets and natural hazy images for training and uses an attention mechanism to increase dehazing performance. These methods can reduce the dependence of the dehazing network on the data set and have a more robust generalization.

## 3. Methods

**Figure 2:**
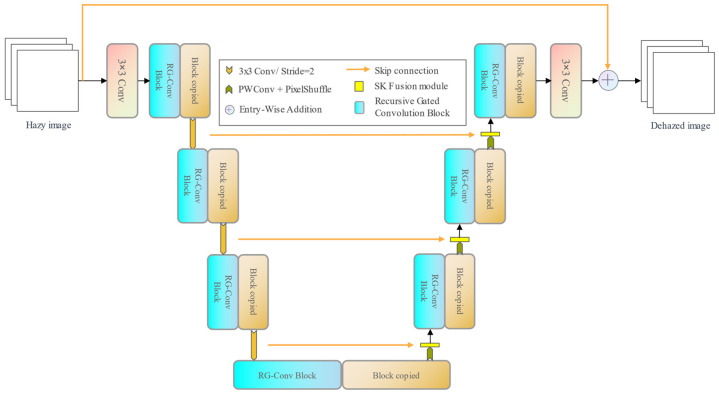
The overall architecture of DRGNet.

The Dehaze Recursive Gated U-Net (DRGNet) is a 7-stage adaptation of the U-Net, depicted in Figure 2. Each stage of the DRGNet comprises one or more stacked Recursive Gated Convolution Blocks (RG-Conv Blocks), which employ the recursive gated convolution. We recognized the limitations of the original recursive gated convolution for image dehazing tasks. As such, we implemented several enhancements. We also introduced SK fusion modules, derived from the SK module, and can dynamically fuse features from the encoder and decoder while introducing an attention mechanism to the model.

### 3.1. Motivation

With the development of deep learning technology and the deepening of people’s understanding of image dehazing tasks, dehazing models based on deep learning are performing better and better in image dehazing tasks. However, as illustrated in Figure 1, we have observed that these high-performing dehazing models often come with complex architectures, numerous parameters, and significant computational overhead. Such complexity in models is detrimental to training, inference, and deployment. Therefore, a lean model with excellent dehazing performance is necessary and holds significant research value.

Owing to the haze effect precipitated by atmospheric scattering, the transmission of light is disrupted by airborne particulates, causing specific pixel values in the image to darken. Channels vary in their response to light scattering and absorption, resulting in differential darkening effects. A typical method in the Prior-based approach is the DCP method, positing differences in pixel value distribution across channels in a hazy image window. To some extent, the DCP method’s success verifies the effectiveness of channel-by-channel feature extraction. Recursive gated convolution operates by executing gated convolutions recursively on the input information’s channel dimension to extract features. Intuitively, superior to traditional convolution, recursive gated convolution more efficiently merges features from different channels, thereby enhancing image dehazing efficiency. The direct application of original recursive gated convolution to image dehazing is challenging, leading to our adaptation of it to cater to image dehazing task specifics. U-Net is an encoder–decoder convolutional neural network design characterized by simplicity, fewer parameters, fast inference speed, and proficiency in image detail information. Combining recursive gated convolution with U-Net architecture offers a lean, high-performance image dehazing network. Attention mechanisms have gained significant attention in recent years. Our study has revealed that by making subtle modifications to the SK module, it is possible to introduce an attention mechanism to the model while dynamically fusing feature maps from different branches.

### 3.2. Recursive Gated Convolution Block

**Figure 3:**
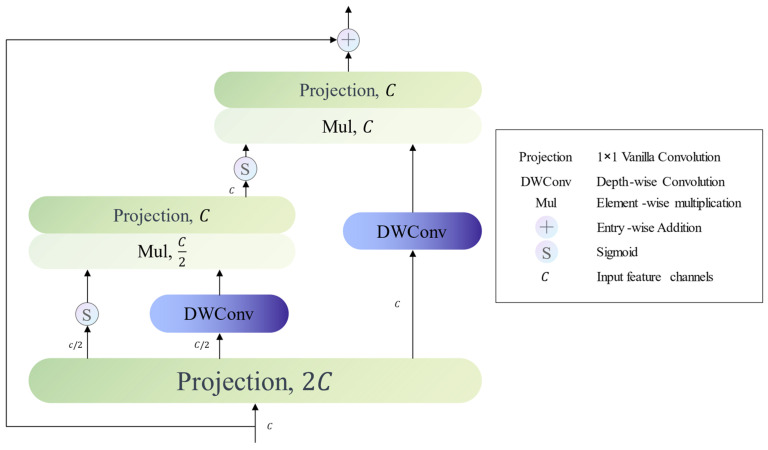
Recursive Gated Convolution Block with second-order interactions.

We propose that the Recursive Gated Convolution Block (RG-Conv Block) is fundamentally rooted in the recursive gated convolution mechanism. Unlike conventional convolution blocks, the RG-Conv Block improves model performance by explicitly modeling high-order interaction mechanisms. Figure 3 depicts the structure of an RG-Conv block with a second-order interaction. Depending on the circumstances, we can easily adjust the value of the order, thereby improving the model’s capability.

Let’s define $X\in R^{HW\times C}$ as the input feature map and C represents the channel number of X. Initially, we leverage a projection layer to effect a dimensional transformation on X. Subsequently, the projection layer’s output is divided by channel dimension, leading to the generation of a series of features:

(2) $$\left[P_{0}^{HW\times C_{0}},D_{0}^{HW\times C_{0}},D_{1}^{HW\times C_{1}},\ldots ,D_{n-1}^{HW\times C_{n-1}}\right]=Proj\left(X\right)\in R^{HW\times 2C}$$

In Equation (2), n symbolizes the value of the order while the channel number $C_{k}$ of each feature adheres to the following conditions: $C_{0}+\sum_{k=0}^{n-1}C_{k}=2C$ and $C_{k}=C/2^{n-k-1}$.

We undertake gated convolution [40] in a recursive style. For an RG-Conv block featuring n-order interaction, the execution of gated convolution is necessary n times. We assume that the output of k-th gated convolution is $P_{k}$. The computation process of $P_{k+1}$ is in line with the Equation (3):

(3) $$P_{k+1}=\phi_{k}(Sigmoid({g_{k}(P}_{k}))⊙f_{k}(D_{k}))\in R^{HW\times C_{k+1}}$$

In this context, $f_{k}$ stands for Depth-wise Convolution [41] and ⊙ represents element-wise multiplication, with $g_{k}$ and $\phi_{k}$ being piecewise functions. In Equation (4), $Linear\left(C_{k-1},C_{k}\right)$ denotes the mapping of the channel dimension of the feature vector from $C_{k-1}$ to $C_{k}$. Equation (5) is the same.

(4) $$g_{k}=\left\{\begin{array}{c} Identity,\;k=0\\ Linear\left(C_{k-1},C_{k}\right),\;k>0 \end{array}\right.$$

(5) $$\phi_{k}=\left\{\begin{array}{c} Identity,\;k+1<n\\ Linear\left(C_{k},C\right),\;k=n-1 \end{array}\right.$$

It is worth mentioning that we performed pertinent ablation experiments in the experimental section. The findings demonstrated that bounded functions such as the Sigmoid function [42] and the Hard-Sigmoid function [43] could effectively circumvent gradient explosion. On the contrary, eliminating the activation function or utilizing unbounded functions such as the ReLU function [44] leads to gradient explosion, while employing the Tanh activation function could also trigger gradient explosion. This likelihood escalates with increasing order n.

### 3.3. SK Fusion Module

The SK module is an attention module that can significantly improve the performance of the model when applied to basic computer vision tasks [45,46]. The SK fusion module we used is a variation of the SK module.

**Figure 4:**
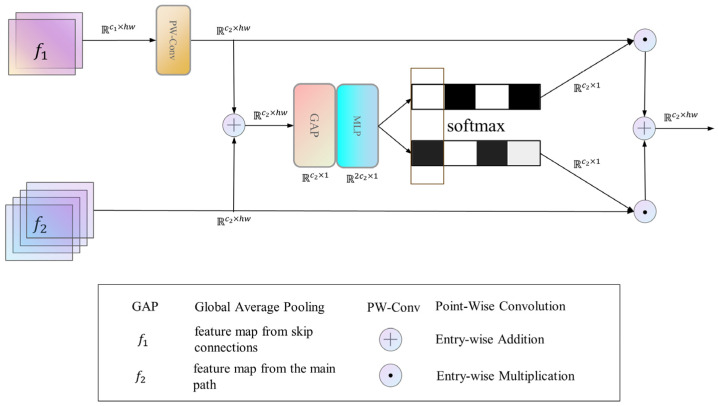
The process of SK Fusion Module fuses various branches. R represents the dimension information of different feature vectors.

In DRG-Net, as depicted in Figure 2, there are skip connections between the encoding and decoding stages, and the features of the encoding and decoding stages are fused to help the network better restore the detailed information. We use the SK fusion module to fuse features from different stages dynamically. For the feature map $f_{1}\in R^{c_{1}\times hw}$ from the encoding stage and the feature map $f_{2}\in R^{c_{2}\times hw}$ from the decoding stage, their process of dynamic fusion by the SK fusion module is shown in Figure 4.

For feature map $f_{1}$, it undergoes a linear function $g\left(f_{1}\right)=Linear\left(c_{1},c_{2}\right)$, which matches its channel dimensions with $f_{2}$. Subsequently, $g\left(f_{1}\right)$ and $f_{2}$ are fused through element-wise addition to generate a feature map denoted as s. Following this, global average pooling [47] is employed to generate channel-wise statistics, multi-layer perceptrons and the softmax function are then used to obtain channel attention for the feature map s, and the channel dimension separates the result to procure the fusion weights of separate branches. The above process can be described by Equation (6).

(6) $$\left\{w_{1},w_{2}\right\}=Split\left(Softmax\left(MLP\left(GAP\left(g\left(f_{1}\right)+f_{2}\right)\right)\right)\right)$$

Lastly, feature maps from distinct branches are added to the fusion weights in a weighted manner to produce the final output of the SK fusion module:

(7) $$Out=w_{1}g\left(f_{1}\right)+w_{2}f_{2}$$

### 3.4. Training Loss

In the training process of DRGNet, we elected to use the L1 loss function. The principal distinction between the L1 and L2 loss (MSE loss) functions lies in their handling of errors. The L2 loss function penalizes more significant errors more severely (as errors are squared), making the model inclined to minimize these substantial errors. However, this may also make the model excessively sensitive to noise or outliers. In image dehazing, such sensitivity could lead the model to overcorrect hazy effects, resulting in the loss or over-sharpening of image details. In contrast, the L1 loss function yields superior results in image dehazing tasks. In the ablation experiments in the subsequent chapters, we also verified this point of view.

### 3.5. Network Architecture Details

DRGNet is a 7-stage derivative of the U-Net blueprint. To simplify the model and ensure performance and stability during training, DRGNet’s architecture details are as follows: each stage accommodates RG-Conv blocks at a quantity ratio of {1:1:1:2:1:1:1}. RG-Blocks in the same stage should have the same interaction order, and the interaction orders of RG-Block in different stages are: {1, 2, 2, 3, 2, 2, 1}, while the kernel size of DW-Conv within each RG-Block is 5. To rigorously evaluate the dehazing capabilities of DRGNet, we engineered three depth-differentiated variants: DRGNet-T, DRGNet-B, and DRGNET-L. Table 1 delineates the architectural details of each variant. In subsequent ablation experiments we will verify the rationality of this design.

## 4. Experimental

### 4.1. Data Set and Experimental Setup

Experiments were conducted on the RESIDE [48] and Haze-4K [49] datasets to validate our method. RESIDE, with its diverse data sources and image content, is subdivided into ITS (Indoor Training Set), OTS (Outdoor Training Set), and SOTS (Synthetic Objective Testing Set). We utilized ITS (13,990 image pairs) and OTS (313,950 image pairs) to train the dehazing network and the indoor and outdoor scenes from the SOTS dataset (500 image pairs each) for testing. The Haze4K dataset, a synthetic dataset, includes 4000 paired images from both indoor and outdoor scenes. A total of 3000 pairs were employed for training and the remaining 1000 for testing.

We employed the PyTorch framework for coding and ran the training and testing on an A100 (80 G) graphics card. During training, the input image dimension was set to 256 × 256. The mini-batch size for variants of DRGNet at varying depths was 32, with 1000 epochs of training. We found that the size of the learning rate significantly influences training stability; hence, we opted for smaller learning rates for deeper variants. For the T, B, and L variants, according to the linear scaling rule [50], we established learning rates of {12 × 10^−4^, 8 × 10^−4^, 4 × 10^−4^}, respectively. We incrementally elevated the learning rate using a warmup strategy for 50 epochs. Subsequently, the cosine decay strategy [51] is employed to gradually reduce the learning rate to 1/100 of the initial value. In the training process, we utilized the AdamW optimizer [52].

### 4.2. Quantitative Comparison and Qualitative Analysis

To corroborate the efficacy of our proposed method, we executed a quantitative performance comparison between the DRGNet and the baseline methods. The results are in Table 2. In this quantitative comparison, the DRGNet performed well on the extensive RESIDE and more concise Haze4K datasets. Compared to existing methods, the DRGNet strikes a balance between performance and model complexity. For example, when compared with the classic dehazing network FFA-Net, the DRGNet-T only used about 1% of the MACs and around 20% of the parameters, and improved PSNR [53] and SSIM [53] by 2.47 and 0.005, respectively. Furthermore, when contrasted with the more advanced MixDehazeNet-S, the DRGNet-T demonstrated a comparable dehazing performance while reducing the MACs and parameters by approximately 76% and 47%, respectively.

**Figure 5:**
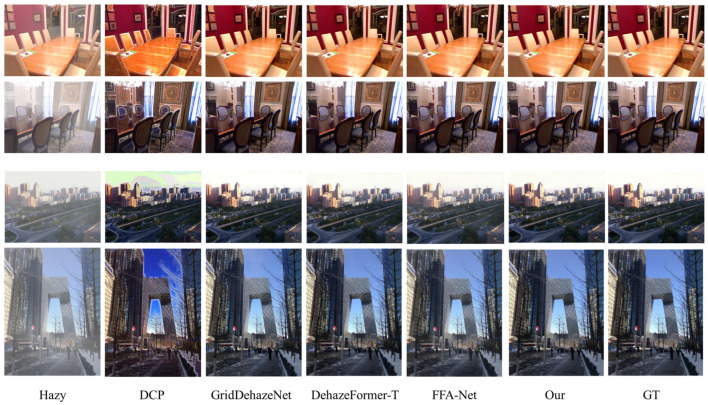
Qualitative comparisons on RESIDE-IN and RESODE-OUT datasets. The first two rows are indoor images, the last two rows are outdoor images, and the last column represents the corresponding ground truth.

Figure 5 showcases the visual dehazing outcomes of the DRGNet-T compared with other methods. Our method outshines existing methods in terms of dehazing quality. The DRGNet mitigates haze influence, resulting in amplified clarity, enhanced contrast, and restored color images. It proficiently retains details and textures camouflaged by the haze, ensuring aesthetically pleasing outputs. Moreover, our network performs robustly when processing hazy images from various scenes, consistently delivering reliable dehazing results. It efficiently mitigates haze while suppressing artifacts, representing an optimal compromise between these two aspects of image restoration.

### 4.3. Ablation Study

**Figure 6:**
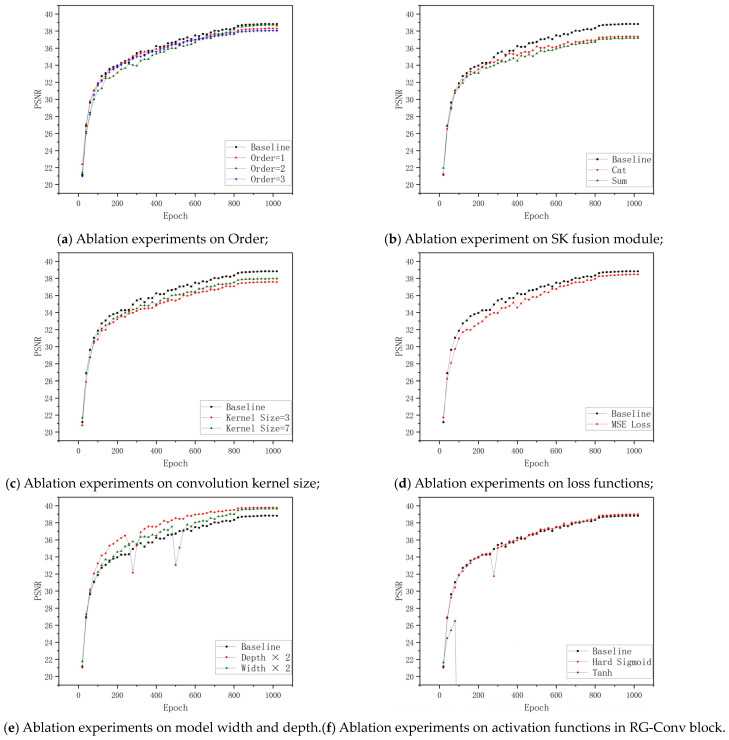
Ablation Study: Training DRGNet-T on SOTS Indoor Dataset. The X-axis represents the number of training iterations, and the Y-axis represents the dehazing performance of the model (we use the Peak Signal-to-Noise Ratio (PSNR) to measure if PSNR = 0 indicates that Nan appeared during the training process). For better visualization, we calculate the average every twenty points and plot it.

To analyze the model’s key components, we conducted ablation studies. Table 3 shows the results of ablation experiments, with Figure 6 showing the training process of the DRGNet-T in selected ablation experiments.

In the DRGNet, we used a series of recursive gated convolution blocks with different orders to facilitate feature extraction. When the order was 1, the recursive gated convolution block was degraded to a standard gated convolution block. Our ablation studies showed performance differences between these two mechanisms, suggesting that the recursive gated convolution mechanism significantly improves the model’s performance. Furthermore, we examined the activation function design within the recursive gated convolution blocks. We incorporated a sigmoid function into the traditional recursive gated convolution mechanism to prevent gradient explosion. Subsequent ablation studies confirmed the effectiveness of our approach: on removing the bounded function or resorting to unbounded functions such as ReLU, NaN values predictably emerged during the training phase. Lastly, we explored the influence of order. The experimental results show that the order can affect the model’s performance and efficiency. If the order is too small, it will affect the model’s performance. If the order is too large, it will become difficult to train and infer the model. Therefore, deploying blocks in different orders across stages at different depths is a feasible strategy, which we adopt.

It is crucial to highlight that the RG-Conv block introduces a notably significant increase in latency. We conjecture that this could stem from the recursive gating mechanism, which engages DW-Conv at each recursion step. Conventional deep learning frameworks often enhance computational efficiency through optimization libraries (e.g., cuDNN) and hardware acceleration (e.g., GPUs). Nonetheless, these optimizations might not seamlessly support DW-Conv in specific scenarios, resulting in suboptimal efficiency.

The original SK Module boasts a lightweight nature, seamlessly integrating without imposing substantial additional computational costs. Conventional U-Net models commonly resort to concatenation fusion for amalgamating features sourced from diverse branches. However, within the DRGNet, we utilized the SK Fusion module, resulting in a conspicuous elevation in the model’s dehazing prowess. The improvement may be attributed to the following factors: (1) The SK fusion module introduces an attention mechanism to the model. (2) Unlike concatenation fusion, the SK fusion module can dynamically fuse features from different branches.

The kernel size refers to the convolution kernel size in the DW-Conv of the recursive gated convolution block. Our experiments revealed that kernel size 5 is an optimal choice for the DRGNet-T. However, any increase or decrease in kernel size led to a significant drop in performance.

The experimental results show that compared with the L2 loss function, the improvement brought about by using the L1 loss function is significant. Simultaneously, the simplicity of the L1 loss function also reduces the computational cost and latency of the model.

Irrespective of whether the depth or width of the DRGNet-T is augmented, a significant enhancement in the model’s performance ensues. This observation underscores the robust scalability inherent in the DRGNet. It is noteworthy that, when compared to broader networks, deeper networks exhibit an enhanced performance. However, this advantage comes at the expense of increased parameters and computational demands. Simultaneously, as the network depth expands, the count of RG-Conv blocks also rises, resulting in a substantial escalation of latency in deeper networks.

## 5. Discussion

This paper introduces an efficient dehazing network: the DRGNet. We use the U-Net with local and global residuals as the basic architecture to extract multi-scale information of features. To enhance the extraction of features from various channels in hazy images, we have improved the recursive gated convolution mechanism and created RG-Conv blocks using it.

Simultaneously, we have leveraged SK fusion to supersede the conventional cascade fusion. This innovation empowers the model’s fusion layer to dynamically integrate feature maps from distinct branches while incorporating an attention mechanism into the model’s framework.

We have presented three distinct variants of the DRGNet, varying in depth, and evaluated their performance across multiple publicly available datasets. The test results unequivocally showcase the exceptional capabilities of the DRGNet. We conducted extensive tests to confirm the effectiveness of our proposed designs. This will provide valuable guidance for future researchers to analyze the key designs that enhance the performance in dehazing networks.

## Figures and Tables

**Table 1:** Architecture details of DRGNet with varying depths. “Depth” indicates the number of blocks per stage, “Order” indicates the interaction order of blocks per stage.

Name	Depth	Order
DRGNet-T	{2, 2, 2, 4, 2, 2, 2}	{1, 2, 2, 3, 2, 2, 1}
DRGNet-B	{4, 4, 4, 8, 4, 4, 4}	{1, 2, 2, 3, 2, 2, 1}
DRGNet-L	{8, 8, 8, 16, 8, 8, 8}	{1, 2, 2, 3, 2, 2, 1}

**Table 2:** Benchmarking Dehazing Techniques on Three Datasets. We use a single A100 (80 G) graphics card to train our dehazing network. For a fair comparison, we used a single RTX3090 graphics card to test the overhead of DRGNet. Data for other methods in the table are taken from their respective papers. ‘-’ indicates that there are no such data in the original paper.

Model	RESIDE-IN		RESIDE-OUT		Haze4K		Overhead		
	PSNR	SSIM	PSNR	SSIM	PSNR	SSIM	MACs	Param	Latency
DCP [22]	16.62	0.818	19.13	0.815	14.01	0.760	-	-	-
DehazeNet [25]	19.82	0.821	24.75	0.927	19.12	0.840	0.581 G	0.009 M	0.919 ms
MSCNN [26]	19.84	0.833	22.06	0.908	14.01	0.510	0.525 G	0.008 M	0.619 ms
AOD-Net [27]	20.51	0.816	24.14	0.920	17.15	0.830	0.115 G	0.002 M	0.390 ms
GCANet [28]	30.23	0.980	-	-	25.09	0.923	18.41 G	0.702 M	3.695 ms
GridDehazeNet [29]	32.16	0.984	30.86	0.982	-	-	21.49 G	0.956 M	9.905 ms
MSBDN [30]	33.67	0.985	33.48	0.982	22.99	0.850	41.54 G	31.35 M	13.250 ms
PFDN [31]	32.68	0.976	-	-	-	-	50.46 G	11.27 M	4.809 ms
FFA-Net [32]	36.39	0.989	33.57	0.984	26.96	0.950	287.8 G	4.456 M	55.91 ms
PMNet [33]	38.41	0.990	34.74	0.985	-	-	81.13 G	18.90 M	28.08 ms
UDN [34]	38.62	0.991	34.92	0.987	-	-	-	4.25 M	-
gUNet-T [35]	37.99	0.993	34.52	0.983	31.60	0.984	2.595 G	0.805 M	3.391 ms
MixDehazeNet-S [36]	39.47	0.995	35.09	0.985	-	-	22.06 G	3.16 M	14.56 ms
DehazeFormer-T [37]	35.15	0.989	33.71	0.982	-	-	6.658 G	0.686 M	10.59 ms
MAXIM [54]	38.11	0.991	34.19	0.985	-	-	216 G	14.10 M	-
SGID-PFF [55]	38.52	0.991	30.20	0.975	-	-	152.80 G	13.87 M	20.92 ms
LKD-B [56]	38.57	0.993	34.81	0.983	-	-	12.20 G	1.22 M	-
DEA-Net [57]	40.20	0.993	36.03	0.989	33.19	0.99	32.23 G	3.653 M	7.093 ms
DRGNet-T	38.86	0.994	34.81	0.983	32.42	0.986	2.907 G	0.939 M	7.57 ms
DRGNet-B	39.82	0.995	35.32	0.984	32.89	0.987	5.207 G	1.675 M	13.70 ms
DRGNet-L	40.76	0.996	36.33	0.986	33.21	0.988	9.803 G	3.146 M	25.77 ms

**Table 3:** Ablation study of network architectures.

Methods	RESIDE-IN		Overhead		
	PSNR	SSIM	MACs	Param	Latency
Baseline	38.86	0.994	2.907 G	0.939 M	7.57 ms
RGC → GC	38.34 ↓	0.993 ↓	2.607 G ↓	0.806 M ↓	6.21 ms ↓
Sigmoid → Hard-Sigmoid	39.02 ↑	0.995 ↑	2.909 G ↑	0.939 M	7.67 ms ↑
→ ReLU	NaN	NaN	2.907 G	0.939 M	7.81 ms ↑
→ GeLU	NaN	NaN	2.907 G	0.939 M	7.43 ms ↓
Order → [1, 1, 1, 1, 1, 1, 1]	38.34 ↓	0.993 ↓	2.607 G ↓	0.806 M ↓	6.21 ms ↓
→ [2, 2, 2, 2, 2, 2, 2]	38.76 ↓	0.994	3.036 G ↑	0.918 M ↓	7.54 ms ↓
→ [3, 3, 3, 3, 3, 3, 3]	38.07 ↓	0.991 ↓	3.181 G ↑	0.951 M ↑	9.26 ms ↑
SK Fusion → Cat	37.59 ↓	0.990 ↓	3.115 G ↑	0.958 M ↑	7.31 ms ↓
→ Sum	37.32 ↓	0.989 ↓	2.904 G ↓	0.934 M ↓	7.10 ms ↓
Kernel Size = 5 → 3	37.64 ↓	0.989 ↓	2.69 G ↓	0.904 M ↓	7.11 ms ↓
→ 7	38.00 ↓	0.991 ↓	3.239 G ↑	0.991 M ↑	7.67 ms ↑
L1 LOSS → L2 LOSS	38.49 ↓	0.993 ↓	2.909 G ↑	0.939 M	7.81 ms ↑
Depth × 2	39.82 ↑	0.995 ↑	5.207 G ↑	1.675 M ↑	13.70 ms ↑
Width × √2	39.70 ↑	0.995 ↑	4.965 G ↑	1.638 M ↑	7.35 ms ↑

## Data Availability

Experimental data can be found in the following link: https://github.com/AquaLaker/Efficient-Dehazing-with-Recursive-Gated-Convolution-in-U-Net-A-Novel-Approach-for-Image-Dehazing (accessed on 6 September 2023).
